# Blood concentrations of the cytokines IL-1beta, IL-6, IL-10, TNF-alpha and IFN-gamma during experimentally induced swine dysentery

**DOI:** 10.1186/1751-0147-50-32

**Published:** 2008-08-12

**Authors:** Robert Kruse, Birgitta Essén-Gustavsson, Caroline Fossum, Marianne Jensen-Waern

**Affiliations:** 1 Department of Clinical Sciences, Section for Comparative Physiology and Medicine, Swedish University of Agricultural Sciences, P.O. Box 7054, S-750 07, Uppsala, Sweden; 2 Department of Molecular Biosciences, Section of Veterinary Immunology and Virology, Swedish University of Agricultural Sciences, P.O. Box 7054, S-750 07, Uppsala, Sweden

## Abstract

**Background:**

Knowledge of the cytokine response at infection with *Brachyspira hyodysenteriae *can help understanding disease mechanisme involved during swine dysentery. Since this knowledge is still limited the aim of the present study was to induce dysentery experimentally in pigs and to monitor the development of important immunoregulatory cytokines in blood collected at various stages of the disease.

**Methods:**

Ten conventional pigs (~23 kg) were orally inoculated with *Brachyspira hyodysenteriae *B204^T^. Eight animals developed muco-haemorrhagic diarrhoea with impaired general body condition. Blood was sampled before inoculation and repeatedly during acute dysentery and recovery periods and cytokine levels of IL-1β, IL-6, Il-10, TNF-α and IFN-γ were measured by ELISA.

**Results:**

IL-1β was increased at the beginning of the dysentery period and coincided with the appearance of Serum amyloid A and clinical signs of disease. TNF-α increased in all animals after inoculation, with a peak during dysentery, and IL-6 was found in 3 animals during dysentery and in the 2 animals that did not develop clinical signs of disease. IL-10 was found in all sick animals during the recovery period. IFN-γ was not detected on any occasion.

**Conclusion:**

*B. hyodysenteriae *inoculation induced production of systemic levels of IL-1β during the dysentery period and increased levels of IL-10 coincided with recovery from dysentery.

## Background

Swine dysentery is an important disease caused by the spirochete *Brachyspira hyodysenteriae *[1]. This infection is confined to the large intestine and results in muco-haemorrhagic diarrhoea, deterioration of general condition and a high mortality if untreated [2]. We have previously reported on the increase of numbers of neutrophils, monocytes and CD8α+ lymphocytes during dysentery and the increase in γδ T cells and *B. hyodysenteriae*-specific antibodies during the recovery period [3,4]. The knowledge of the cytokine response during swine dysentery is still limited and most of the information available comes from *in vitro *studies [5-7]. However, considering the complexity of a natural infection, in which a multitude of factors are involved, *in vivo *findings are imperative for understanding various clinical responses to an infection.

Locally produced cytokines may reach concentrations that are systemically detectable during infections. Increased amounts of pro-inflammatory cytokines generally have a negative influence on the growth and well-being of the animal [8,9]. However, many cytokines are of major importance for enhancing the innate immune response and directing the adaptive immunity against either a Th1 or Th2 dominated response [10,11]. The pro-inflammatory cytokines IL-1β, TNF-α and IL-6, which are readily induced by the presence of lipopolysaccharides from Gram-negative bacteria [12] play an important role in the synthesis of acute phase proteins and often participate in the pathogenesis of many infections [13]. In this context IL-1β is a key cytokine that is produced by many porcine cells, such as macrophages and intestinal epithelial cells [14]. Macrophages are also major producers of TNF-α and this cytokine is also commonly expressed during infections. IL-6, in addition to its pro-inflammatory role, is considered to be a cytokine of importance for the development of an antigen-specific humoral response during some infections [15]. IL-10 is an important anti-inflammatory cytokine, which downregulates the production of pro-inflammatory cytokines and generally protects the animal from systemic inflammation (for review see [16]). IL-10 is primarily produced by Th2 cells, monocytes, B cells [17,18], and as IL-1β, it is also produced by intestinal epithelial cells [14]. IFN-γ is an activator of the cytotoxic T cell pathway and may be of interest during swine dysentery in view of the increase in CD8α+ T and/or NK cells that has previously been reported to occur during dysentery [3,4,19].

The aim of the present study was therefore to induce dysentery experimentally in pigs and to monitor the development of some immunoregulatory cytokines (IL-1β, IL-6, Il-10, TNF-α and IFN-γ) in blood collected at various stages of the disease.

## Methods

### Animals and housing

The Ethical Committee for Animal Experiments, Uppsala, Sweden, approved the experimental design.

Ten clinically healthy crossbreed pigs (Yorkshire × Swedish Landrace) were obtained from a conventional piglet-producing herd, with a well-known health status and free from swine dysentery, and were kept in the experimental facilities at the Department of Clinical Sciences, SLU, Uppsala, Sweden. The experimental facilities were localised 10 km from the closest pig herd and had not been in use for at least 6 months prior to arrival. In addition, all personnel that handled the pigs had no contact with other farm animals during the experimental period. The pigs were of both sexes, 8–10 weeks old and had an average weight of 13 kg (range 11–16 kg) at arrival. All animals were housed individually, had free access to water and were fed twice a day with a commercial finisher diet without growth promoters (Singelveg^®^SPK, Lantmännen, Stockholm, Sweden). The animals were given 26 days to acclimatise. During this acclimatisation period, straw bedding material was used. At arrival, faecal samples were taken from all pigs and analysed for the presence of parasite eggs, *Brachyspira spp., Salmonella spp. *and *Yersinia spp. *All pigs were found to be free of these pathogens. Clinical health examinations, including rectal body temperature each morning, were performed daily on all animals throughout the study and the animals were weighed at least once a week for calculation of their daily weight gain. In order to avoid the effect of different growth rates during the pig's individual fattening period, the daily weight gain was divided by the animal's live weight and presented as daily weight gain per kg live weight.

### Experimental design

After the acclimatisation period a provocative feeding regime was used to facilitate onset of infection [20]. Briefly, four days prior to inoculation and during the three following days of oral inoculation, every second meal was replaced by pure soybean meal. In addition, the straw bedding material was replaced by synthetic fur blankets during the experimental period in order to minimise fibre ingestion from the straw that could have interfered with the infection model. From the day of inoculation and onwards, all animals were moved in-between the pens once a day. The inoculum consisted of 30 mL/day (90 mL in total) of brain-heart infusion (BHI) broth containing approximately 10^7^-10^9^*B. hyodysenteriae *strain B204^T ^(ATCC 31212)/mL. The bacteria were propagated as described by [21] and prior to inoculation the bacterial growth, motility and purity were evaluated by phase contrast microscopy. The total experimental period lasted for 65 days. Day 1 of the swine dysentery period refers to the first day of the diarrhoea period, and day 1 of the recovery period refers to the day when a change from diarrhoea to normal or just slightly loose faeces occurred. All animals were euthanised with an overdose of pentobarbital sodium. Eight pigs developed swine dysentery and were euthanised 19 to 23 days after the first signs of recovery, while two remained clinically healthy and were euthanised 35 days after inoculation. A necropsy was performed on all animals.

### Sampling of faeces and blood

Faecal samples were collected with rectal swabs from all animals once a week throughout the study, and in addition dysentery-affected animals were sampled once a day during days with clinical signs of disease. The faecal samples were examined for shedding of *Brachyspira spp*. as described by [21]. Blood samples were collected into tubes without additives from the jugular vein of all animals before the soybean diet and the inoculum were given (pre-inoculation). Pigs that developed dysentery were also sampled once a day during the first 4 days with clinical signs, and at days 1, 3, 7 and 11 of the recovery period. Pigs without any clinical signs of disease were sampled at days 4, 14, 21, 28 and 35 post-inoculation. All blood samples were centrifuged at 1500 × g, after which serum was collected and stored at -80°C until further analysed.

### Serum amyloid A (SAA) assays

SAA was measured in sera with commercially available ELISA kits (Tridelta Phase range SAA kit, Tridelta Development Limited, Greystones, Wicklow, Ireland).

### Cytokine assays

Serum concentrations of Il-1β, IL-6, IL-10, TNF-α and IFN-γ were determined in duplicates with commercially available ELISA kits for detection of porcine cytokines (Quantikine Porcine Immunoassays, R&D systems Europe Limited, Abingdon, UK). The minimum limits of detection were as follows: IL-1β 10 pg mL^-1^; IL-6 10 pg mL^-1^; IL-10 1.8 pg mL^-1^; TNF-α 2.8 pg mL^-1^and IFN-γ 2.7 pg mL^-1^.

### Statistical analyses

**Figure 1:**
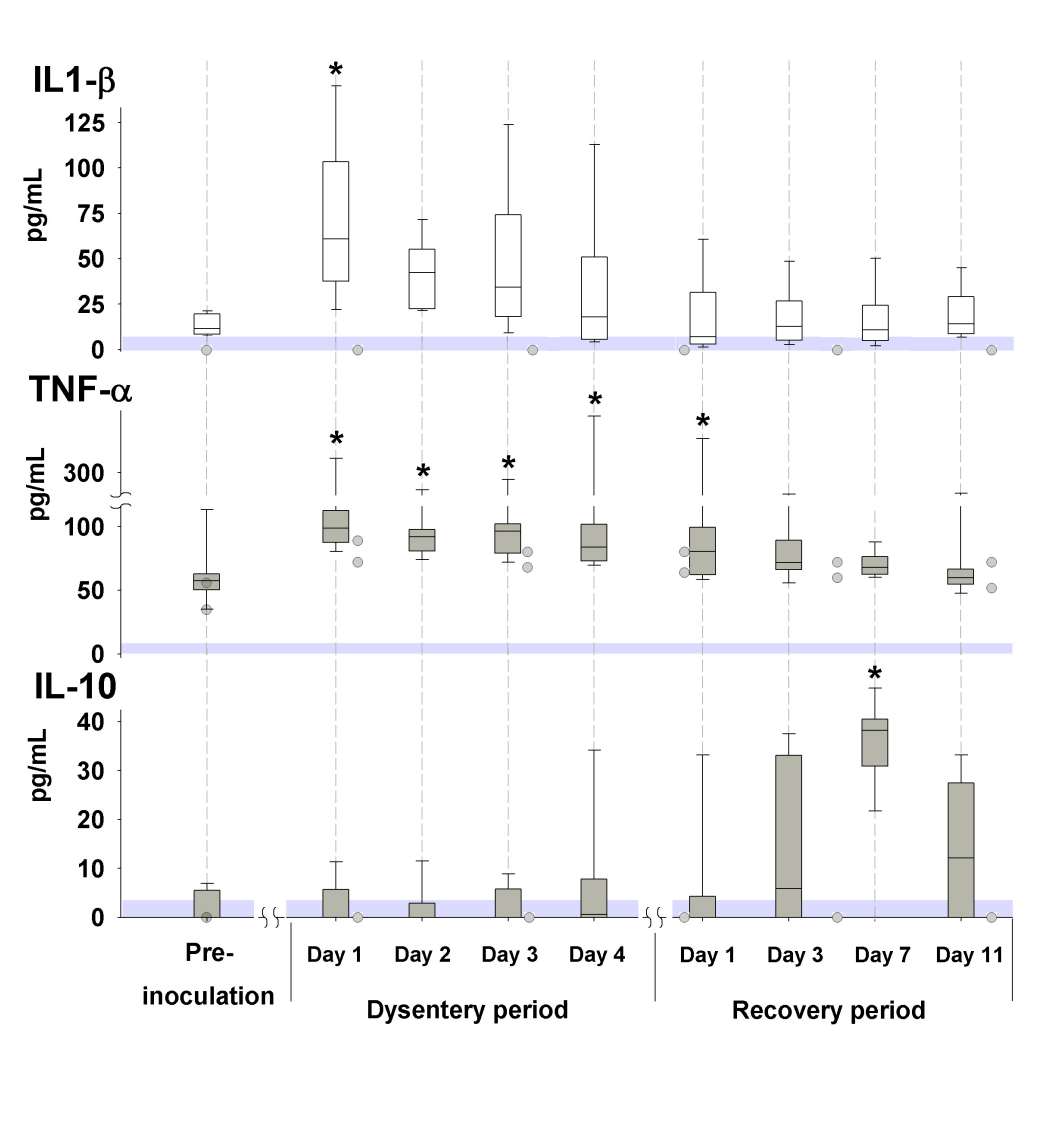
**Serum concentrations of cytokines during experimentally induced swine dysentery**. Serum concentrations of IL-1β, TNF-α and IL-10 before inoculation, during clinical signs of dysentery, and during the recovery period are shown by the box plot. The shaded circles illustrates the individual values of the two animals that remained clinically healthy sampled before inoculation and at days 4, 14, 21, 28 and 35 post-inoculation. The shaded areas above zero represent the detection limit of the assays. * significantly different from the pre-inoculation value.

Data are presented in the text as mean ± SD. The boundaries of the box plot in figure 1 indicate the 25th percentile, the median value and the 75th percentile, whereas the whiskers indicate the 95th and 5th percentiles. One pig had to be euthanised on the second day of clinical signs because of the severity of the disease and is therefore missing from later sampling points, leaving a total of 7 dysentery-affected animals. In addition, there are three missing samples from different pigs during recovery and therefore the means at day 7 are from 5 out of 7 animals and at day 11 they are from 6 out of 7 animals. To compare differences between measurement times, analysis of variance (ANOVA, Holm-Sidak Method) for repeated measures was performed with SigmaStat software (SPSS Science, Chicago, USA). The data were regarded as significantly different at p < 0.05.

## Results

After an average incubation period of 17 days (range 7–31 days) 8 of the 10 inoculated pigs developed dysentery with muco-haemorrhagic diarrhoea. Dysentery was evident for an average of 7 days (range 3–17 days). A deterioration in the general appearance coincided with the muco-haemorrhagic diarrhoea, which occurred for an average of 4 days (range 3–6 days), before signs of recovery were observed. All but one of the dysentery-affected animals recovered spontaneously and showed no major changes in body temperature. The exception, a severely affected animal, had an elevated body temperature (40.6°C) prior to euthanasia and necropsy confirmed the clinical diagnosis, showing severe colitis. All animals shed *B. hyodysenteriae *during the dysentery period. Five of the 7 pigs that recovered stopped shedding, 8 days on average, after the diarrhoea had ended, but two were still shedding at euthanasia. Apart from the clinical signs of dysentery there were no other signs of disease. Two pigs remained clinically healthy throughout the study and they had no diarrhoea or shedding of *B. hyodysenteriae *on any occasion. The necropsies of the 7 animals that recovered and of the 2 pigs that remained clinically healthy did not reveal any significant pathological findings.

The two animals that did not develop clinical signs of dysentery had a steady average daily weight gain per kg live weight of 25 ± 2 g kg^-1 ^throughout the study. The dysentery-affected animals had a daily weight gain per kg of 24 ± 1 g kg^-1 ^prior to the clinical signs of disease. None of these pigs gained weight during the period with clinical signs of dysentery, but during the recovery period their daily weight gain increased to 20 ± 0 g kg^-1^. The concentrations of the acute phase protein SAA in serum collected from the diseased pigs increased from 21 ± 13 mg L^-1 ^before inoculation to 231 ± 187 mg L^-1^, 154 ± 90 mg L^-1 ^and 137 ± 82 mg L^-1^, respectively, during the first three days with clinical signs of disease and then decreased to 25 ± 10 mg L^-1^on the fourth day of dysentery. At days 1 and 7 of the recovery period the SAA levels were 15 ± 7 mg L^-1 ^and 4 ± 3 mg L^-1^, respectively.

The pro-inflammatory cytokine IL-1β was increased in all clinically ill animals on the first day of the dysentery period (Figure. 1). The 2 inoculated pigs without any clinical signs of disease had no detectable or very low levels of IL-1β (Figure. 1) after inoculation. TNF-α increased after inoculation in all clinically ill animals (Figure. 1) with similar increases in the 2 inoculated animals that remained clinically healthy (Figure. 1). IL-10 increased during recovery from disease in all animals with the highest mean value (36 ± 10 pg mL^-1^) observed at day 7 of the recovery period (Figure. 1). IL-10 was absent in all samples from the 2 animals that did not develop dysentery (Figure. 1). Further, low levels of IL-6 (30–40 pg mL^-1^) were detected in 3 pigs during dysentery and recovery, and in the 2 animals that remained clinically healthy similar levels were noted after inoculation (25–30 pg mL^-1^). No IFN-γ values above the lower limit of detection of the assays were observed on any occasion in any animal.

## Discussion

The results of the present study show that experimental swine dysentery induces detectable levels of some key cytokines in the blood and that they vary regarding to the stage of the disease in which they first appear. The experimental model was successful and the hall-marks for this disease, e.g. muco-haemorrhagic diarrhoea, the shedding of *B. hyodysenteriae *in the faeces and the results from the necropsies confirm that the animals were suffering from swine dysentery. In addition, the high bio security at the experimental facility and the absence of clinical signs of disease during the long acclimatisation period further underscores that no overt co-infection was present during the experimental period.

IL-1β is commonly referred to as an endogenous pyrogen and is generally associated with pyrexia. However, swine dysentery does not in general appear to induce fever and in the present study the only animal with an elevated body temperature was the one with the most severe signs of dysentery. Bacterial LPS and endotoxins are common inducers of IL-1β and when these are extracted from *B. hyodysenteriae *they have been shown to induce IL-1β *in vitro *[5,6]. This supports the likelihood that these bacterial compounds contribute to the systemic IL-1β response recorded in the present experimental model. Locally, IL-1 can cause an increase in vascular permeability and oedema, and together with TNF-α it can potentiate the effects of prostaglandins and thereby alter the ion transport in the intestinal epithelial cells in pigs [22]. Hence the effects of IL-1β could play a major role in the development of diarrhoea. IL-1β is known to cause neutrophilia [23] and as reported earlier, increased levels of circulating neutrophils were observed in these animals during dysentery [3] and an increased neutrophil counts has been seen in colon lesions in pigs with dysentery [24,25]. Further, IL-1β has been shown to induce metabolic alterations [26] and may influence the catabolic processes that supply large amounts of glucose to immune cells during clinical signs of disease. Several important gluconeogenic amino acids, such as alanine and glutamine, were observed to decrease in serum in these pigs during clinical signs of dysentery [27].

An increase in TNF-α was noted in serum from all animals after inoculation of *B. hyodysenteriae*, with a peak during clinical signs of disease. TNF-α was detected irrespective of the subsequent health status and may therefore reflect a response to the introduction of the novel *B. hyodysenteriae *antigen or a subsequent subclinical co-infection with *B. hyodysenteriae *or other bacteria, such as *E. coli*. In addition, even though all animals were free from clinical signs of disease and had normal levels of circulating leucocytes and no significantly elevated levels of SAA before inoculation, low levels of TNF-α concentrations were seen before inoculation. Similar levels of TNF-α have been observed previously in clinically healthy pigs [28,29]. Expression of TNF-α can be induced in a variety of porcine cells, especially in macrophages/monocytes, in response to LPS and other bacterial cellwall products [30] and systemic levels of TNF-α have been observed in response to *E. coli *infections in pigs [28,31]. In studies on LPS and endotoxin extracts obtained from *B. hyodysenteriae *discrepancies regarding TNF-α induction have been observed [5,6,32]. The TNF-α ELISA that were used in the present study detects both free and the more stable receptor-bound TNF-α and is therefore unable to distinguish between the two forms. Free TNF-α, which is the biologically active form, is rapidly cleared from plasma and thus, the prolonged increase of TNF-α after inoculation might be influenced by the build up of soluble receptor-bound TNF-α. Nonetheless, the presence of TNF-α during the dysentery period may influence the pathogenesis since it can facilitate lesion development and the gastrointestinal tract is known to be sensitive to the presence of this cytokine [33].

IL-6, mainly produced by cells such as Th2 cells, monocytes, B cells and muscle tissue [34], is often regarded as a useful biomarker of bacterial infections in pigs, and has been reported to be present for several days after an *Actinobacillus pleuropneumoniae *infection [35]. This is partly due to its slow and stable plasma kinetics (for review see [36]). Elevated serum levels of IL-6 have been observed in pigs after injection with LPS and endotoxin extracts from *B. hyodysenteriae *[32]. In the present study it seemed that serum IL-6 was not a reliable marker of swine dysentery, as only three of the sick animals showed detectable levels of this cytokine in the blood during dysentery. Still, it cannot be excluded that the other pigs also produced IL-6 on an occasion other than those covered by the sampling frequency. Both the presence of IL-6 and TNF-α after inoculation in the two animals that remained clinically healthy could indicate a subclinical infection with *B. hyodysenteriae*.

The appearance of the anti-inflammatory cytokine IL-10 during the recovery period was associated with the disappearance of clinical signs of disease and the return of IL-1β and TNF-α to pre-inoculation values. The increase in IL-10 during the recovery period is likely to be an important factor for normalisation of the elevated monocyte, neutrophil and CD8α+ lymphocyte levels that occurs during recovery [3]. Further, the recovery period and the presence of IL-10 in the blood of the sick animals coincided with the appearance of *B. hyodysenteriae*-specific serum antibodies [3]. This may be due to the stimulatory effect of IL-10 on B cells that will enhance antibody production and induce Ig-class switching and plasma cell differentiation [37-39].

IL-1β, TNF-α and IL-6 are all important inducers of hepatic production of SAA (for review see [40], which is a common acute phase protein that has been shown to be clinically relevant in pigs [41]. In the present study the levels of SAA in most animals accordingly rose 30- to 60-fold after the appearance of IL-1β and coincided with clinical signs of dysentery. SAA has several important functions during immune responses, such as enhancement of tissue infiltration of polymorphonuclear cells, monocytes and T cells into the inflamed tissues [42,43] and might thereby enhance cellular migration into the colon during swine dysentery. Even though IL-6 is a potent inducer of SAA, it requires IL-1 in order to be able to act as an inducer [44]. This could explain why the two pigs that remained clinically healthy had no detectable SAA, in spite of the presence of IL-6.

No detectable levels of IFN-γ were present in the blood on any of the sampling occasions in any animal. These negative results do not rule out the possibility that IFN-γ was produced locally in the intestinal tract. This question will be further addressed with microarray analyses of colon biopsies from pigs with dysentery. Considering the increase of monocytes in these animals [3] during dysentery and the importance of IFN-γ for monocyte activation [45], the involvement of IFN-γ at some stage of this disease seems plausible. Further, the increase in CD8+ lymphocytes [3] during dysentery may also contribute to the speculation of IFN-γ involvement, since CD8+ cells are important producers of IFN-γ [46]. It has been shown that porcine peripheral blood lymphocytes from pigs vaccinated with *B. hyodysenteriae *antigen produce significant levels of IFN-γ in response to *in vitro *stimulation with the same antigens as were used during the vaccination [7]. However, this IFN-γ response was not found in lymphocytes from colonic compartments of the pigs, suggesting that there are compartmental differences. It is important to consider that this may also be true for the production of cytokines other than IFN-γ.

## Conclusion

In conclusion, increased levels of the pro-inflammatory cytokine IL-1β and SAA were detected during the period with dysentery, whereas an increase in IL-10 was seen during the recovery period. Further experimental studies are needed to better understand the immune mechanisms that protect against swine dysentery, but the results of the present study indicate that key cytokines are involved systemically and not only confined to local areas of the affected colon.

## Authors' contributions

RK and MJW designed the study design and performed the experimental infection. RK were responsible for the acquisition, analysis and interpretation of data. RK and MJW have been involved in drafting the manuscript, and BEG and CF have been involved in revising it critically and contributed intellectually.
